# Supplementary material for: Towards Whole Health Toxicology: In-Silico Prediction of Diseases Sensitive to Multi-Chemical Exposures
Source: Toxics. 2022 Dec 8;10(12):764. doi: 10.3390/toxics10120764 (PMC9784704; doi:10.3390/toxics10120764)
Supplement: Supplementary file 1 [file toxics-10-00764-s001.zip › toxics-1996410-SI.pdf]

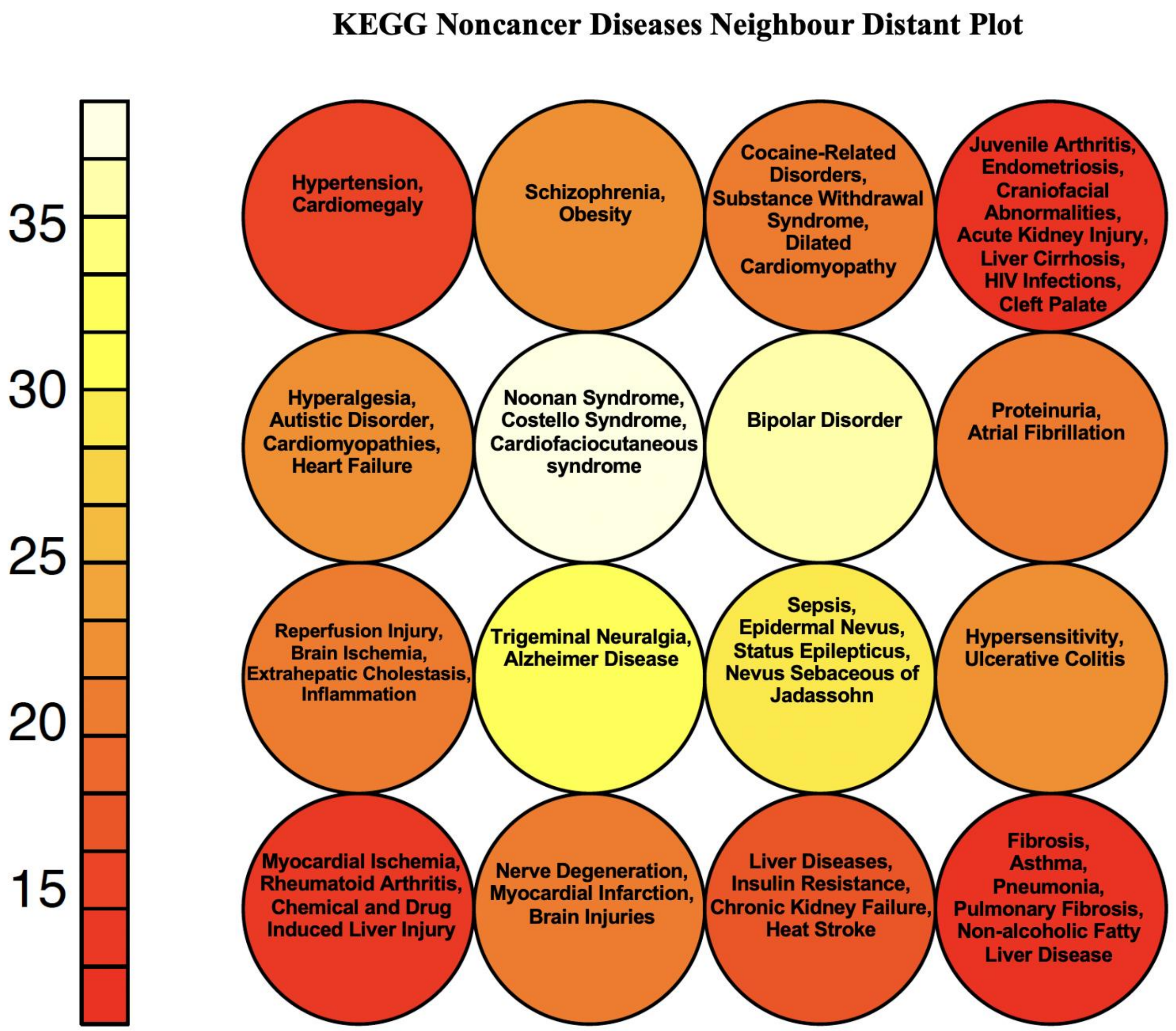

Figure S1. SOM neighbor distant plot of non-neoplastic diseases based on the similarity of their overlap with chemically sensitive KEGG pathways.

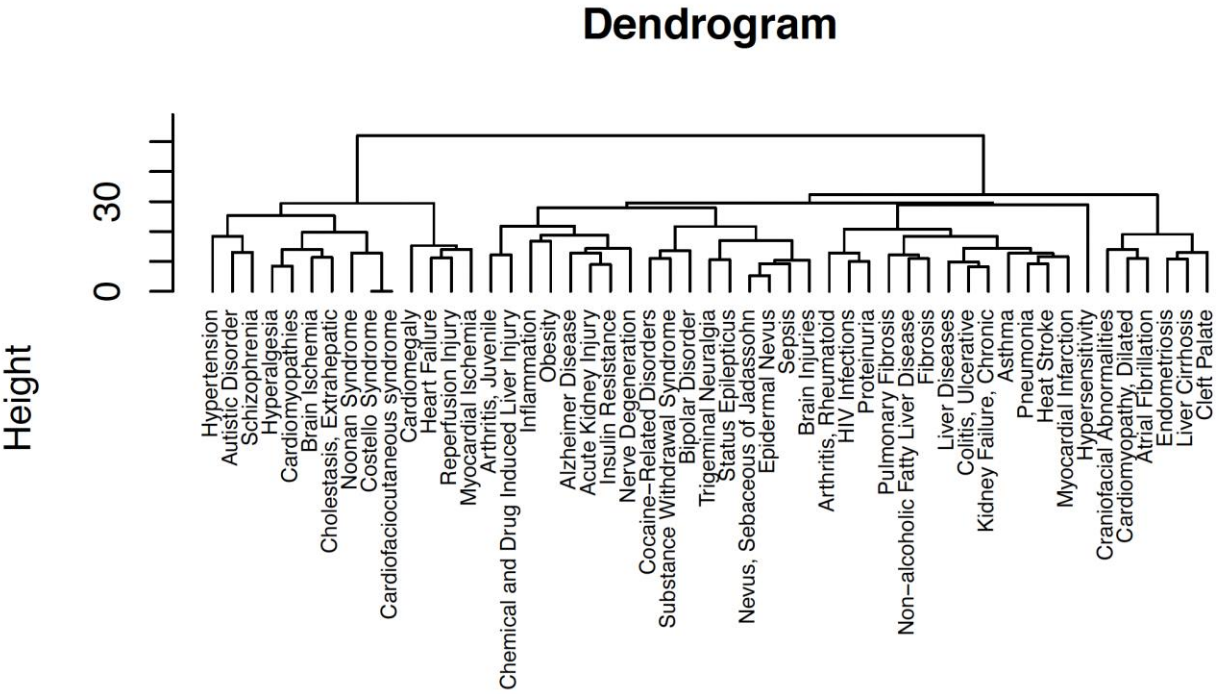

Figure S2. Hierarchical clustering of non-neoplastic diseases based on the similarity of their overlap with chemically sensitive KEGG pathways.
